# Spatiotemporal database of US congressional elections, 1896–2014

**DOI:** 10.1038/sdata.2017.108

**Published:** 2017-08-15

**Authors:** Levi John Wolf

**Affiliations:** 1 Spatial Analysis Research Center, School of Geographical Sciences and Urban Planning, Arizona State University. 975 S. Myrtle Avenue, Tempe, Arizona 85281, USA; 2 Center for Spatial Data Science, University of Chicago. 5737 S. Ellis Ave, Chicago, Illinois 60630, USA

**Keywords:** Politics, Geography, Government

## Abstract

High-quality historical data about US Congressional elections has long provided common ground for electoral studies. However, advances in geographic information science have recently made it efficient to compile, distribute, and analyze large spatio-temporal data sets on the structure of US Congressional districts. A single spatio-temporal data set that relates US Congressional election results to the spatial extent of the constituencies has not yet been developed. To address this, existing high-quality data sets of elections returns were combined with a spatiotemporal data set on Congressional district boundaries to generate a new spatio-temporal database of US Congressional election results that are explicitly linked to the geospatial data about the districts themselves.

## Background & Summary

Longitudinal study of Congressional elections in the United States is not new. With the publication of King^1^, high-quality data on US Congressional elections at constituency level was made available for various studies of redistricting, voter behavior, and electoral system analysis. For instance, a series of influential studies on the impact of redistricting on the electoral system uses this data^2,3^. In the decade after it was published, many other studies of American elections also used this data.

However, later studies of elections have not provided data to extend King^1^ directly. In most cases, these studies both extend and enrich the original data set, providing a superset of the original Congressional elections data. Often, these analyses focused on sociodemographic study of redistricting’s impact on various aspects of the electoral system^4–6^ or are general studies of the social and demographic structure of American Congressional geography^7,8^. While privately-owned data exists for this purpose, the price of obtaining coverage comparable to (ref. 1) is high. Thus, the Constituency-Level Electoral Archive (CLEA) was developed in part to provide an extended, more detailed, and open data set on legislative electoral geography^9^. For US Congressional elections, this data set also provides much more data about minor parties and candidates themselves, and has been used in a variety of contemporary electoral studies^10–13^. Since the CLEA is a multi-country data set, it is used often for comparative studies that examine the extent to which particular theories about politics or polimetric techniques hold across electoral contexts. For US elections, the CLEA has been used for longitudinal analysis of electoral structures, examining how specific electoral properties, like effective number of political parties or competitiveness, change over time.

For the geospatial research on US Congressional elections, work has focused on the development and propagation of macro-scale sectionalism and the construction of ideological and geographic voting blocs over time^14–16^, as well as the analysis of scale-sensitive political alignments^17,18^. Recently, calls for a revitalized quantitative electoral geography, focusing on electoral systems analysis and voter behavior, have been made^19,20^, and many foundational problems in the spatial analysis of electoral systems, such as those articulated by Gudgin and Taylor^21^, have been explicitly reclaimed by contemporary authors^22,23^. This has seen an explosion of spatio-temporal analysis of the electoral geography of the US Congress^24,25^, as well as analyses at non-Congressional spatial scales^26–28^, and non-American electoral systems^29,30^. Altogether, the literature on electoral analysis has become both robust and wide ranging.

Complementing the revitalization of electoral systems analysis, most of the data generated in this literature has been openly shared under permissive licenses. However, the construction and maintenance of spatially-referenced data sets for Congressional analysis can be a more difficult process than analysis of elections at a state or county level. Indeed, King^1^ and the CLEA only provide spatial information in terms of the states in which districts are found. They do not provide information about the shape or extent of the districts, nor the neighborhood & topological relations between districts. Geography & spatial effects, are much richer than nesting relationships alone, however, so longitudinal study of spatial effects in congressional districts is quite restricted^31^. While the CLEA provides a selection of ‘georeferenced elections data’ (the GRED), this data set is not comprehensive, with limited temporal scope when compared to US elections coverage in the CLEA. To remedy this, I have constructed a general-purpose spatial database that extends King^1^ forward in time using data from the CLEA and novel data on incumbency. With this extended dataset, I connect the individual district geometries compiled by Lewis *et al.*^32^ to yield a single spatio-temporal database of US Congressional elections. In what follows, I discuss the process for constructing this data set, compare the relative values of the source data sets, and briefly discuss potential use cases or novel analyses that this new data may provide.

## Methods

To extend King^1^ using the CLEA and bind both to Lewis *et al.*^32^, a common key across all data sets was needed. This required a coherent data modeling strategy that could encompass the abstractions in each of the data sources. Thus, the data set I construct is a collection of the results of general elections to the US Congress. Elections to a Congress are composed of some number of *contests*, which are electoral challenges in which some number of winning candidates are declared. Each contest occurs within some territorially-bound constituency, or *district*, which may or may not be unique in each Congress. Relating the three data sets required disambiguating the relationship between contests, districts, and the Congresses. To do this, the Inter-University Consortium for Political and Social Research (ICPSR) numerical codes for states used in King^1^ were converted to US Census Bureau Federal Information Processing Standard Codes (FIPS codes). These codes are contained in column state_fips of the example table segment shown in Table 1.

Then, a composite database key was constructed to refer uniquely to a contest’s Congress, state, and district. The first three characters of the composite index reflect the Congress number of the record, with zero-padding on the left if the Congress number is less than three digits. The second three digits of the composite index are the zero-padded state FIPS code. The third three digits of the composite index are the zero-padded district number. Finding a common district indexing scheme was the first design challenge for this data set. In all cases, the sources used different conventions to refer to ‘at-large’ districts, districts that are the entire state. In the CLEA, at-large districts were variously referred to as the first district (vermont 01), as the ‘zeroth’ district (vermont 00), or having no number (vermont). In all cases, Lewis *et al.*^32^ referred to at-large districts as the ‘zeroth’ district. In both data sets, district numbers refer to the spatial constituency in which the contest occurs, so multiple contests may have the same district number. In contrast, King^1^ constructs multiple district indexes in the case of a multi-member district. In at-large contests, the district index is labeled decreasing from 98. So, if two candidates run at-large in a state, their district numbers are 98 and 97. In general, this means that a single district, in the sense of a territorial entity, may have multiple indices if there is more than one contest in the district.

The Lewis *et al.*^32^ convention is the most simple and robust for this application since it treats the district consistently as a spatial object, rather than as a hybrid of contest and district concepts. In addition, the Lewis *et al.*^32^ indexing strategy retains an advantage of King’s^1^ index, since at-large contests can be separated efficiently. At-large contests can be structurally different from typical Congressional elections that occur at the sub-state level, and at-large occasionally merit separate consideration. So, the CLEA records were made consistent with the Lewis *et al.*^32^ convention, and the King^1^ records were converted to this convention as well. The final index is contained in the geom_id column of Table 1, and the original indices retained in king_dist and lewis_dist, which reflect the two consistent styles of district numbers.

In addition, a unique index for the geometries themselves, the index from Lewis *et al.*^32^, is retained in each record. This is composed of four three-digit codes. The first component is the zero-padded state FIPS code. The second component is the Congress in which the district shape first appeared. The third component is the last Congress in which the district shape was used. The final component reflects the district number assigned to the district during its lifetime. This index uniquely identifies the geometries of constituencies in US Congressional elections, whereas the other index provides a unique identifier for the contests, referring to their Congress, state, and constituency. In addition, this index allows for the construction of a high-quality redistricting indicator variable since a contest in a ‘new’ district is one whose congress variable matches the second triplet in the component index.

For elections before 1992, the vote_share and turnout, delsouth, and inc covariates are taken directly from King^1^. For later elections, vote_share, delsouth, and turnout is constructed from the CLEA and inc is coded by hand. All variables aim to replicate the method used to generate King^1^. The simplest to replicate is delsouth, a binary variable indicating that a record is in a southern state. The vote shares, in this case, are the share of the two-party vote cast for Democratic candidates. To construct this from the CLEA, the total number of votes cast for the candidate endorsed by the major party candidates is recorded. Thus, in cases of ‘fusion voting,’ where the same candidate appears on multiple party tickets, these counts are added to the major party’s total. The sum of these votes is turnout. Then, the share of turnout that the Democratic candidate receives is the vote_share. To match the structure of King^1^, the detail in the party identification in the CLEA was reduced to three parties: Democrat, Republican, and Other. In most cases, the reduction in parties was not significant. However, one case should be mentioned: Farmer-Labor candidates before the Democrat Farmer-Labor merger in 1944 were considered to be Democrats. This decision does not affect the resulting data product, since only King^1^ was used during this period, but will be apparent in the validation plots shown below. The CLEA does not contain incumbency information, so the inc variable was derived by hand from Congressional rosters. The post-1992 inc variate was coded to match King^1^: a Republican incumbent who runs for reelection is coded as -1, a Democrat who runs for reelection is coded as 1, and a zero is recorded when there is no single incumbent. Together, this comprises the dataset produced from the CLEA. It provides similar data to King^1^ in addition to extending past 1992 and enriching the data with spatial information. The period of overlap in King^1^ and the data derived form the CLEA, all US elections from 1896 to 1992, will be analyzed in the validation section to ensure that the CLEA data after 1992 comports with the data sourced from King^1^.

After this, all data was inserted into a SQLite database and a outer join conducted, retaining all district shapes. The join resulted in over 98% of matches on keys, so only a tiny fraction of the districts in Lewis *et al.*^32^ did not find matches in the extended King^1^. Most of the remaining missing entities reflected duplications, malformed original entries that slipped through the data cleaning process, or non-voting constituencies that were not recorded in the CLEA or King^1^. The geometry information was stored in a text format in a column, wkb, of the resulting comma-separated table. This column is shown truncated in Table 1, and contains Polygons or MultiPolygons (as defined by Open Geospatial Consortium^33^) encoded in well-known binary (WKB), stated in hexidecimal. This more concise statement of WKB-encoded geometry is common in database software (such as PostGIS), but still results in a column with long elements. The coordinates are stored without a coordinate system using the NAD83 datum, inherited from Lewis *et al.*^32^.

### Code availability

The methods used to generate the data set will be made available through the Open Science framework. All scripts were implemented in Python, and requires a few Python data analysis libraries: pandas, a tabular data processing library, geopandas, a geospatial tabular processing library, numpy, a numerical computation library, and SQLite, used for the final out-of-core database join. In addition, a makefile is provided for convenience, to ensure the build process executes in the correct order.

When run in the correct order, the scripts generate intermediate data and final data products from a collection of sources. First, Kollman *et al.*^9^, King^1^, and the manually-constructed collection of incumbency information for elections beyond 1992 are contained in a sources directory. The first script, 00_get_all_shapes.py, collects all district shapes from the repository maintained by Lewis *et al.*^32^, placing them in the sources directory as well. Two intermediate data products are constructed. First, after running 01_data_munge_clea.py, a cleaned and party-reduced version of the CLEA data is stored in intermediates/clean_clea.csv. Second, the next script in the sequence, 02_rebuild_database.py, combines all of the district shapes together in a single table in a SQLite database. Then, the fourth script, 03_extended6311.py, concatenates the original King^1^ with the cleaned CLEA data. This first product, the extended_6311.csv data set, has the same schema as the final data set constructed by 04_final_merge.py, which accesses the SQLite database and merges the extended King^1^ with the collection of district shapes. In this merge, two final outputs are generated, products/pre1948.csv and products/post1948.csv, which split the results of the merge in two parts. The split divides the series roughly in half, and corresponds to the divsiion between full Congresses organized by the Legislative Reorganization Act of 1946. Finally, if if more columns from the Lewis dataset are required by analysts, the retained columns of the merge in 04_final_merge.py can be changed without affecting the merge process.

## Data Records

As discussed in the Code Availability, three data products are combined within Data Citation 1. The spatial dataset due to Lewis *et al.*^32^ is split into pre-1948 and post-1948 components to reduce the size of the resulting product. To join these pieces together, the latter table’s header must be removed and the tables concatenated. Both tables have the schema discussed above in Table 1 with columns in the same order. To assist those who have no need for the spatial referencing, products/extended_6311.csv, named for the original ICPSR numerical designation of King^1^, contains the complete elections data with the geometric column omitted.

## Technical Validation

**Figure 1:**
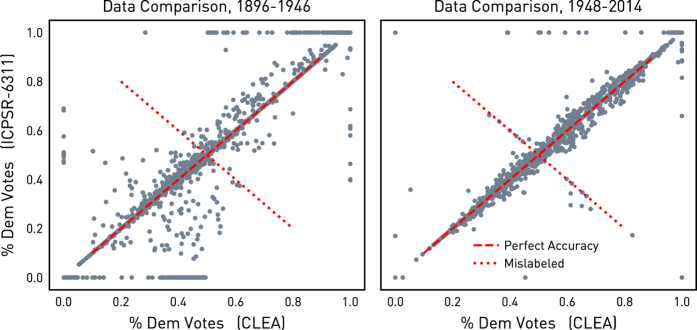
Relationship between Democrat share of the two-party vote in King^1^ (ICPSR-6311) and that constructed from Kollman *et al.*^9^ (CLEA).

After merging and validation, the resulting two-party vote shares constructed from the CLEA were compared to the original source King^1^ duting the period of overlap in Data Citation 1, from 1896 to 1992. In this period, the CLEA data is not retained in the final product. But, comparisons over this period of overlap illustrates how closely the method constructs a dataset from the CLEA with the same semantics as King^1^. First, the comparison shown in Fig. 1 presents the scatterplot of the Democratic party vote shares in Congressional elections constructed using the CLEA and that from King^1^ (ICPSR-6311). The plot for the early period of overlap is shown on the left, and the comparison over the latter period is shown on the right. The early period of overlap contains all Congresses conducted before the Legislative Reorganization Act of 1946, and the latter period contains all full Congresses that take place after the passage of the Act. The correspondence in the two data sets is high, but is much better in the second half of the data than in the first. This is likely both due to the way the Democrat-Farmer-Labor faction was processed and the relative disappearance of minor factional party classifications in the CLEA in the period after 1948. In addition, the prevalence of fusion voting declines in this period, which makes tabulation of the two-party vote much simpleer. Thus, this comparison indicates that using the data derived from the CLEA should provide an accurate post-1992 extension of King^1^, since accuracy is better for the contemporary Congresses.

However, this plot does clearly show cases where the CLEA and King^1^ are almost perfectly negatively correlated. When isolated, these cases occurred when the two source data sets disagreed about the party identification of the legislators in a contest. Upon further examination, these cases were determined to be errors in the CLEA and were reported. However, since these coding errors are detected only in the pre-1992 portion of the CLEA-derived data, these few coding errors do not propagate into the derived spatiotemporal database.

**Figure 2:**
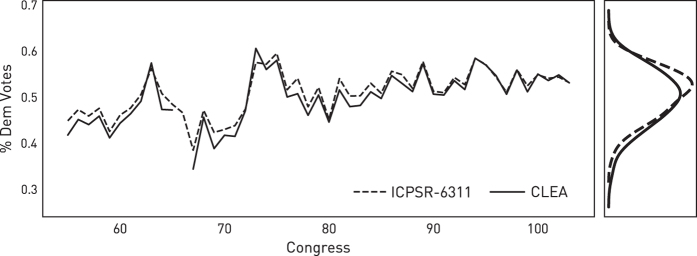
Comparisons of the national average Democratic vote share between King^1^ (ICPSR-6311) and Kollman *et al.*^9^ (CLEA).

A second verification step, comparing the share of votes Democrats receive and the share of seats Democrats win in the US Congress between the two data sets was conducted. This comparison is shown in Fig. 2. Both sources generate similar estimates in these two cases, and again tend to track better in later Congresses. Notably, however, the two most recent CLEA releases (versions 8 and 9) omit elections in the United States for 1918, which this graph makes clear. Since the derived dataset uses King^1^ for all years before 1992, this missing data does not affect the final data product. Thus, with these two comparisons, it seems the two-party vote data generated from the CLEA is sufficient to extend King^1^ past 1992, and the final spatio-temporal database of US Congressional elections since 1896 is coherent.

## Usage Notes

To use this enhanced version of King^1^, the data must first be loaded correctly into an efficient spatial format. The format chosen here is standards-compliant and can be read by any tabular data reader with access to GDAL, the Geographic Data Abstraction Library. In addition, the table can be read directly into various SQL engines (such as PostgreSQL or SQLite), and the well-known binary column converted directly to geometries using appropriate PostGIS or Spatialite functionality. Then, spatial analysis can be conducted using standard statistical packages^34–36^. This may include spatial econometric analysis of electoral models^37^, exploratory local spatial modeling^24^, or cluster analysis and voter diffusion detection^25^. Alternatively, when combined with further primary and secondary data, this spatio-temporal database may prove useful in the study of a wide variety questions about electoral structures, such as redistricting, sorting, partisan bias, polarization, or sectional analysis.

## Additional Information

**Competing interests:** The author declares no competing financial interests.

**How to cite this article:** Wolf, L. J. Spatiotemporal database of US congressional elections, 1896–2014. *Sci. Data* 4:170108 doi: 10.1038/sdata.2017.108 (2017).

**Publisher’s note:** Springer Nature remains neutral with regard to jurisdictional claims in published maps and institutional affiliations.

## Supplementary Material



## Figures and Tables

**Table 1:**
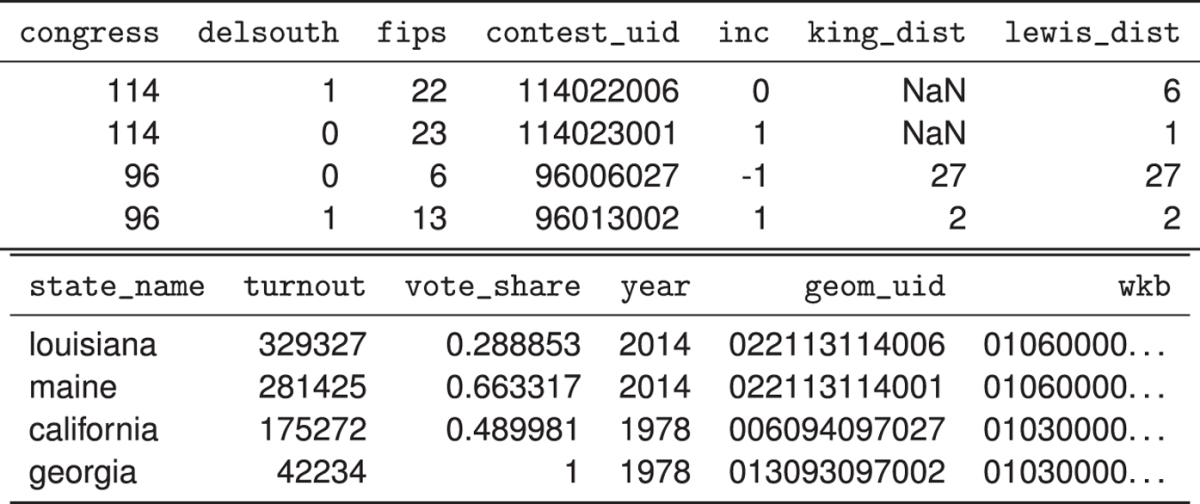
The example schema of the final data product, broken into two lines.
